# Influence of Selected Food Product Groups Consumption Frequency on Acne-Related Quality of Life in a National Sample of Polish Female Adolescents

**DOI:** 10.3390/ijerph191811670

**Published:** 2022-09-16

**Authors:** Julia Rudzińska, Dominika Głąbska

**Affiliations:** Department of Dietetics, Institute of Human Nutrition Sciences, Warsaw University of Life Sciences (WULS-SGGW), 159C Nowoursynowska Street, 02-776 Warsaw, Poland

**Keywords:** diet, dietary behaviours, acne vulgaris, skin, dermatological diseases, quality of life, adolescent, female

## Abstract

Acne vulgaris affects over 80% of adolescents, mainly female ones, and may reduce their general quality of life, so its prevention and treatment are becoming necessary, while among the options for acne management, the potential influence of diet is indicated. The aim of the study was to assess the influence of selected food product groups consumption frequency on acne-related quality of life in a national sample of Polish female adolescents. The studied population of 1370 Polish female adolescents was gathered using random quota sampling of secondary schools representative of the whole country. The acne-related quality of life was assessed using the Acne Quality of Life (AQoL) Scale with the calculated Social Quality of Life (SOCQOL) Score, as well as the Acne Disability Questionnaire (ADQ) with the calculated Cardiff Acne Disability Index (CADI). The acne-related food product groups’ consumption frequency was assessed using the Acne-specific Food Frequency Questionnaire (Acne-FFQ). There were no differences in food product consumption between subgroups stratified by the acne-related quality of life (*p* > 0.05), and there was no correlation between food product consumption and the results of the SOCQOL Score (*p* > 0.05). For the CADI, positive correlations were indicated for fast foods (*p* = 0.0450; R = 0.0688), salty snacks (*p* = 0.0342; R = 0.0727), and chocolate confectionary (*p* = 0.0147; R = 0.0837), while a negative correlation was indicated for dairy beverages other than milk (*p* = 0.0414; R = −0.0701). In the studied group of Polish female adolescents, fast foods, salty snacks, and chocolate confectionary were indicated as potential acne-promoting factors, while dairy beverages other than milk were indicated as a potential acne-protective factor.

## 1. Introduction

Acne vulgaris, also called adolescent acne [1], is a disease of pilosebaceous unit, causing noninflammatory lesions (open and closed comedones), as well as inflammatory lesions (papules, pustules, and nodules) [2], which affects over 80% of adolescents [3]. Acne is initiated during puberty onset (for females at the age of 10–11 years and for males at the age of 11–12). Its incidence peaks at the age of 15 for both females and males, and it declines during puberty completion (for females at the age of 15–17 years and for males at the age of 16–17), but the frequency in females is, for all age groups, higher than for males [4]. However, it may also be diagnosed during adulthood, as either persistent acne (disease continued or relapsed during adulthood), or late acne (incidence during adulthood) [5].

An analysis conducted based on the Global Burden of Disease Study 2019 [6] revealed that the prevalence of acne vulgaris has increased since 1990 by approximately 48% globally, but it also indicated an almost fourfold variation in the age-standardized prevalence rate in various countries—from 15.8 for Poland to 62.7 cases per 1000 people for Luxembourg. However, as the authors indicated a potential problem with undiagnosed and untreated acne, which may have caused underestimation for some countries [6], a Polish study conducted in a representative sample of Polish students indicated an acne prevalence of 75.1% for female and 74.6% for male individuals [7].

Due to its symptoms, acne may influence the everyday life of affected adolescents, including psychosocial and emotional impairment [8,9], reduced daily activities [9], and modified body image [8], as well as a reduced general quality of life [8,9,10,11]. This problem is especially vital, as a number of adolescents even without acne are characterized by a reduced self-confidence, and tend to be negative about themselves and their appearance [12]. As a result, the impact of acne on the quality of life is comparable with the impact of epilepsy, diabetes, and coronary heart disease [13].

Taking into account the significant emotional influence of acne, its prevention and treatment are becoming necessary [14], addressing the main pathological factors associated with its development, including increased sebum production, irregular follicular desquamation, *Propionibacterium acnes* proliferation and inflammation of area [15]. The guidelines of care for the management of acne vulgaris, developed based on a systematic review of the literature, listed potential options for acne management, including the influence of diet (glycaemic index and dairy products) [16]. Similarly, a recent review of the evidence by Dall’Oglio et al. [17] defined potential acne-promoting (high glycaemic index score, dairy products, fat foods, chocolate) and acne-protective factors (specific fatty acids, fruit, vegetable), confirming the possibility of applying dietary prevention and diet therapy for acne.

Considering the described state of knowledge, the aim of the study was to assess influence of selected food product groups consumption frequency on acne-related quality of life in a national sample of Polish female adolescents.

## 2. Materials and Methods

### 2.1. Ethical Issues and Study Design

The study was conducted based on the approval of the Ethics Committee of the Institute of Human Nutrition Sciences of the Warsaw University of Life Sciences (WULS-SGGW) (No. 23/2020), and according to the guidelines of the Declaration of Helsinki, and the study participants and their parents/legal guardians provided their informed consent for study participation.

The study was conducted in the period of May 2021–February 2022, at the Department of Dietetics, Institute of Human Nutrition Sciences, WULS–SGGW, and it included assessment of the influence of selected food product groups consumption frequency on acne-related quality of life in a national sample of Polish female adolescents. The national population-based sample of female adolescents was gathered through recruitment within their secondary schools, a same method to that used by a corresponding study on male adolescents [18].

### 2.2. Studied Sample of Polish Female Adolescents

The studied sample was recruited within a population of Polish adolescents aged 14–20 years, corresponding to the highest acne prevalence [4]. Such an age range is in agreement with the age range of secondary school students in Poland (regular age range of 15–19 years, broadened to 14–20 years, due to a possibility of younger children’s admission to secondary school, and a possibility of extending secondary school education). Taking into account the relatively high Net Enrolment Rate (NER) for Polish secondary education of 89.38% [19], the recruitment was conducted within secondary schools, which is a common procedure in Poland [20]. Such a procedure allows recruitment of a nationally representative group of secondary school students from all regions, including large cities and small towns.

The recruitment was based on the random selection of secondary schools in Poland from the national database of secondary schools, which were chosen in two stages, in order to provide a random quota sampling procedure. Within the first stage, from each voivodeship, being a basic administrative unit in Poland (total number of 16 voivodeships), 5 counties were randomly selected (total number of 80 counties). Within the second stage, from each county, 5 schools were randomly selected (total number of 400 secondary schools).

The principals of randomly selected schools were invited to agree to the participation of students in the conducted study. If they agreed, they informed students about the school’s participation in the study, and the students and their parents/legal guardians received all necessary details and were asked for their informed consent for study participation. 

If students and their parents/legal guardians agreed to study participation, they were provided a link to an online questionnaire, which was anonymously completed. Based on the answers provided, some respondents were excluded from the study if any conditions appeared, which may have influenced the reliability of the results obtained.

The inclusion criteria for the study were formulated as follows:

− Being a student at a secondary school selected within the study;
− Female gender;
− Age of 14–20 years;
− Polish ethnicity.

The exclusion criteria from the study were formulated as follows:

− Any missing answers within the questionnaire;
− Any unreliable answers within the questionnaire.

**Figure 1:**
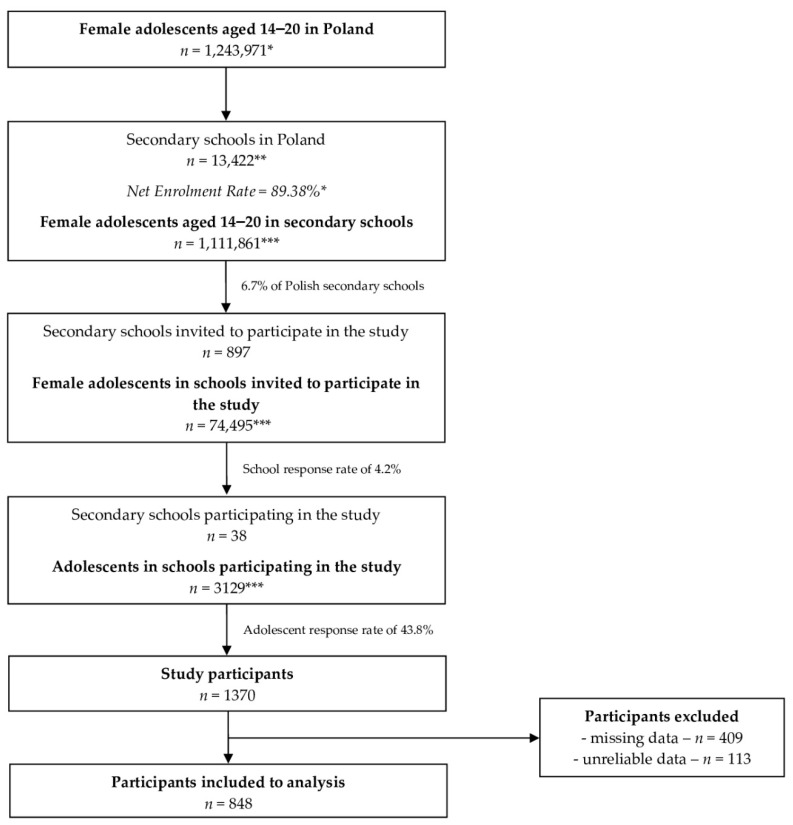
The sampling and participant recruitment process conducted within the study; * data from the Polish Central Statistical Office [19,24]; ** data from the Polish Ministry of National Education [25]; *** calculated on the basis of the data available.

The sampling and participant recruitment process conducted within the study is presented in Figure 1. Finally, the study was conducted in 38 secondary schools, which is a typical school response rate for studies conducted during this period in Poland and is comparable with the school response rate observed by the other authors [21,22]. Such a low response rate is explained by the other authors as a result of the fact that some school teachers or principals prefer not to have any interruptions in their classes or their school not to participate in such studies [23].

A total number of 1370 secondary school female students meeting the inclusion criteria completed the questionnaire, while after exclusion based on the exclusion criteria, a final number of 848 students were included in the analysis. The sample size is in agreement with a previously calculated minimum sample size of 384 (calculated based on a population of Polish female adolescents of 1,440,528 for December 2020 [24], for the level of confidence of 95% and the maximum error of 5%).

### 2.3. Applied Questionnaire

The Computer-Assisted Web Interview (CAWI) method was used within the study, and the Google Forms tool was applied. The online questionnaires were anonymously completed, with neither sensitive nor personal data being gathered, and none of the questions identified any participant of the study. The questions which were included within the questionnaire were associated with verification of the inclusion and exclusion criteria, assessment of the quality of life, and assessment of the selected food product groups consumption frequency. The vast majority of questions were formulated as close-ended ones, while some open-ended ones were included to assess the frequency of consumption of specific products (standard form of questions within the food frequency questionnaire), so the total time needed to complete the questionnaire was estimated as 10 min.

In order to assess acne-related quality of life, two questionnaires were applied: the Acne Quality of Life (AQoL) Scale, which was developed and validated by Gupta et al. [26], and the Acne Disability Questionnaire (ADQ), which was developed by Motley et al. [27] and also validated [28].

The AQoL Scale [26] is based on questions about the extent to which adolescents experience the described feelings as a result of their acne, with 9 items describing feelings included. The feelings are described as: (1) not feeling self-conscious in the presence of others; (2) decrease in socialization with others; (3) difficulties in relationship with partner; (4) difficulties in relationship with close friends; (5) difficulties in relationship with immediate family; (6) feeling like an “outcast” most of the time because of the effect of acne upon appearance; (7) people making fun of appearance; (8) feeling rejected in romantic relationship because of the effect of acne upon appearance; (9) feeling rejected by friends because of the effect of acne upon appearance. The respondent is asked to score each item (described feeling) as: “not at all” (scored as 0), “mildly” (scored as 1), “moderately” (scored as 2), or “very markedly” (scored as 3). Based on the AQoL Scale, the Social Quality of Life (SOCQOL) Score may be calculated as the mean value for 9 items describing feelings, resulting in a total score from 0 to 3 [26].

The ADQ [27,28] is based on questions about the extent to which adolescents experience the described feelings as a result of their acne, and about their opinions on their acne, with 5 items being included. The feelings/opinions on their acne are described as: (1) feeling aggressive, frustrated, or embarrassed; (2) feeling interference in daily social life, social events, or relationships with members of the opposite sex; (3) avoided public changing facilities or wearing swimming costumes; (4) feelings about the appearance of skin; (5) how bad they think their acne is. The respondent is asked to score each item from 0 to 3. Based on the ADQ, the Cardiff Acne Disability Index (CADI) may be calculated as the total value for 5 items, resulting in a total score from 0 to 15 [27,28].

In order to assess acne-related selected food product groups consumption frequency, the Acne-specific Food Frequency Questionnaire (Acne-FFQ) [18] was applied. The Acne-FFQ is based on questions about the frequency of consumption of food products associated with acne incidence and/or course, with 14 food items with potential acne-promoting or potential acne-protective influence being included. The food products are indicated as follows: vegetables, fruit, water, milk, other dairy beverages, white bread, wholegrain bread, other white cereal products, other wholegrain cereal products, fish, fast food, salty snacks, chocolate confectionary, and other confectionary. The respondent is asked to define the number of servings of specific products being consumed during a typical week, with the serving size for each product being described, and the respondent is allowed to declare not only integers, but also decimal parts. The included questions are adapted from the previously validated food frequency questionnaires: Ironic-FFQ to assess intake of products being sources of iron [29], Iodine-FFQ to assess intake of products being sources of iodine [30], and Mg-FFQ to assess intake of products being sources of magnesium [31]. The serving sizes were established based on the Polish atlas of portion sizes of food products and dishes [32].

Due to the fact that the original questionnaires were developed in English, they were translated into Polish and adapted, with the transcultural adaptation to be used in a Polish population. Based on the recommendations by the World Health Organization [33], the whole process was conducted within three stages: (1) forward translation from English to Polish (by native Polish-speaking, English-fluent researcher, with knowledge about discipline), (2) backward translation from Polish to English (by independent English-fluent researcher, not being familiar with the aim of the study and original questionnaire), (3) final polishing of the questionnaire to obtain the accurate transcultural adaptation while ensuring conceptual, semantic, idiomatic, and cultural equivalence, conducted based on all previous version of the questionnaire by an expert panel (of native Polish-speaking, English-fluent researchers, not participating in the previous stages) [33].

### 2.4. Statistical Analysis

The Shapiro–Wilk test revealed nonparametric distributions for the studied variables. Taking this into account, the further analyses were conducted while using the Mann–Whitney U test (to compare groups) and the Spearman rank correlation coefficient (to verify correlations).

The accepted level of significance was *p* ≤ 0.05. Statistica 13.0 (Statsoft Inc., Tulsa, OK, USA) was used for the statistical analysis.

## 3. Results

The general characteristics of the group of Polish female adolescents participating in the study are presented in Table 1. The majority of participants in the study were aged 15–18 years (85.5%) and living in cities of 20,000–100,000 residents (60.1%).

The AQoL Scale detailed results accompanied by the SOCQOL Score for the group of Polish female adolescents participating in the study are presented in Table 2. In spite of the fact that for all AQoL Scale items rather low mean scores were observed, high scores were seen for some respondents, resulting in a maximum score of 3 for both AQoL Scale items and the SOCQOL Score.

The ADQ detailed results accompanied by the CADI in the group of Polish female adolescents participating in the study are presented in Table 3. In spite of the fact that for all ADQ items rather low mean scores were observed, high scores were seen for some respondents, resulting in a maximum score of 3 for ADQ items and a maximum score of 15 for the CADI.

The Acne-FFQ detailed results in the group of Polish female adolescents participating in the study are presented in Table 4. The Acne-FFQ detailed results indicate that the female adolescents participating in the study were characterized by diverse dietary behaviours, as for specific products the declared consumption varied from not consuming at all to high consumption.

The analysis of correlations between the consumption of food items within the Acne-FFQ and the acne-related quality of life, assessed based on the SOCQOL Score calculated from the AQoL Scale results, as well as the CADI calculated from the ADQ in the group of Polish female adolescents participating in the study is presented in Table 5. The analysis revealed no association for the SOCQOL Score (*p* > 0.05). In the case of the CADI, positive correlations were indicated for fast foods (*p* = 0.0450; R = 0.0688), salty snacks (*p* = 0.0342; R = 0.0727), and chocolate confectionary (*p* = 0.0147; R = 0.0837), while a negative correlation was indicated for dairy beverages other than milk (*p* = 0.0414; R = −0.0701).

The comparison of the consumption of food items within the Acne-FFQ in subgroups stratified by the acne-related quality of life, assessed based on the AQoL Scale in the group of Polish female adolescents participating in the study, is presented in Table 6. The analysis revealed no statistically significant differences for the food items assessed within the Acne-FFQ (*p* > 0.05).

The comparison of the consumption of food items within the Acne-FFQ in subgroups stratified by the acne-related quality of life, assessed based on the ADQ in the group of Polish female adolescents participating in the study, is presented in Table 7. The analysis revealed no statistically significant differences for the food items assessed within the Acne-FFQ (*p* > 0.05).

## 4. Discussion

The problem of acne-related quality of life issues is becoming widely recognized, and a number of studies have aimed to assess how skin problems influence the quality of life, both in clinical [34] and research settings [35], with numerous instruments being considered, including those applied in the presented study: the AQoL Scale, developed by Gupta et al. [26], and the ADQ, developed by Motley et al. [27]. 

The AQoL Scale and SOCQOL Score calculated based on it are indicated to be useful to evaluate the association between acne severity and the quality of life, especially in the case of mildly to moderately affected patients [26]. Similarly, the ADQ and CADI score calculated based on it are indicated to be characterized by a high internal consistency, test–retest reliability, responsiveness to change, and significant correlation with other tools [28]. 

However, in the conducted study, both for the AQoL Scale, and for SOCQOL Score calculated based on it, there was no association with the selected food product groups consumption frequency. Similarly, in a previous Polish study, conducted in a population of male adolescents, the influence of dietary behaviours on the acne-related quality of life assessed using the AQoL Scale and ADQ was incompatible, and depending on the applied questionnaire, different results were observed [18]. Such an observation is in agreement with the results of the study by Ilgen and Derya [36], as they stated no statistically significant association between clinical assessment of acne severity as determined using the Global Acne Grading System (GAGS) and AQoL Scale results. Such observations are explained by authors as resulting from the fact that the acne-related quality of life may be affected by factors other than acne severity and the presence of scars themselves and the potential social, personal, emotional, and school-related problems the acne patient may encounter [36]. Taking this into account, it may be indicated that factors included within the AQoL Scale may be broader than those directly related to acne, and as a result, they may not be influenced by diet or consumption of potential acne-promoting and acne-protective factors. This may be confirmed due to the fact that the AQoL Scale, including nine items, is broader than the ADQ, including five items only, as well as the fact that the AQoL Scale assesses feelings associated with acne, while the ADQ assesses feelings associated with acne and its severity.

Within the conducted study, based on the results obtained for the ADQ, fast foods, salty snacks, and chocolate confectionary were indicated as potential acne-promoting factors, while dairy beverages other than milk were indicated as a potential acne-protective factor. Such observations are in agreement with general statements and may confirm the current state of knowledge.

Fast foods, as well as salty snacks, are commonly indicated as being potentially associated with acne development and progression. Within a recent review of the evidence by Dall’Oglio et al. [17], fat foods, including fast foods, were defined as potential acne-promoting products. At the same time, compared with acne-free individuals, those experiencing acne are characterized by a higher burger consumption [37], higher snack and fast food consumption [38], and higher snack and junk food consumption [39]. As a result, highly processed fast food consumption, typical for Westernized countries, is indicated as being associated with the increased risk of acne development [40]. The observations associated with the acne-promoting influence of fast foods and salty snacks may be explained by the content of salt in those products, which is responsible for the general proinflammatory influence [41], dysregulation of the water–electrolyte balance [42], and edema, being indicated among potential acne-promoting mechanisms [43].

Chocolate, as well as the broader category of confectionary, is also indicated as being potentially associated with acne development and progression. In a recent review of the evidence by Dall’Oglio et al. [17], high-glycaemic-index products and chocolate itself were defined as potential acne-promoting products. At the same time, there is an association between amount of consumed chocolate and the number of acne lesions developed [44], as well as a statistically significant increase in the number of acne scores, comedones, and inflammatory papules due to chocolate consumption [45]. The observations associated with the acne-promoting influence of chocolate may be explained by the enhanced corneocyte desquamation and promotion of bacterial colonization of the residual skin surface components, which have been observed to be caused by dark chocolate consumption, which is indicated among potential acne-promoting mechanisms [46]. However, the influence of chocolate may also be attributed to the sugar content, as it may be responsible for increased levels of insulin-like growth factor-1 (IGF-1), IGF-binding protein, and androgen [47,48], as a result affecting keratinocyte proliferation and apoptosis, accompanied by increased sebum production, which is similarly indicated among potential acne-promoting mechanisms [49].

For the dairy beverages other than milk and their influence on acne development and progression, on the contrary, there are some contradictory results. The meta-analysis by Juhl et al. [50] indicated for any dairy product, including milk, yogurt, and cheese, a statistically significant association with the risk of acne development, but the authors indicated that the results should be interpreted cautiously, due to heterogeneity and bias within the included studies. However, a meta-analysis of the observational studies by Aghasi et al. [51] revealed a positive relationship between the consumption of dairy, total milk, whole milk, and low-fat or skim milk and acne occurrence, but no relationship for yogurt and cheese, indicating that yogurt and cheese may not have as much of an acne-promoting effect as the other dairy products. At the same time, a systematic review by Vaughn and Sivamani [52] indicated that in spite of limited evidence, fermented dairy products may be defined as providing benefits for skin health. Such a potentially beneficial influence may result from the role of probiotics, which influence immune system development and functioning and are also applied in the treatment of acne [53].

It should also be indicated that the food products indicated as potential acne-protective factors, namely dairy beverages, may also positively influence general mental health. It was stated that both high-fat and low-fat dairy products are associated with a reduced prevalence of psychological disorders [54]. Similarly, the higher intake of dairy products was indicated to reduce the state-trait anger expression [55]. Last but not least, higher dairy and calcium intake was proven to coincide with lower perceived stress and higher positive mood scores [56].

In spite of the fact that the conducted study revealed novel observations, some limitations must be listed. The study was based on a subjective assessment of the acne-related quality of life, while the acne severity was not studied. Moreover, the questionnaire applied to assess the frequency of consumption of acne-related food products (Acne-FFQ) was not previously validated in the studied population, and, as for all other food frequency questionnaires, there was a risk of overestimation. Last but not least, the study was conducted only in a Polish population, while the results of the studies conducted in the other countries may reveal different observations.

## 5. Conclusions

In the studied group of Polish female adolescents, the analysis revealed that for the CADI, positive correlations for fast foods, salty snacks, and chocolate confectionary existed, while a negative correlation was indicated for dairy beverages other than milk. Fast foods, salty snacks, and chocolate confectionary were indicated as potential acne-promoting factors, while dairy beverages other than milk were indicated as a potential acne-protective factor.

## Figures and Tables

**Table 1:** The general characteristics of the group of Polish female adolescents participating in the study.

Variable	Subgroups	*n*	%
Age (years)	14	31	3.6
	15	174	20.5
	16	227	26.8
	17	168	19.8
	18	156	18.4
	19	76	9.0
	20	16	1.9
Place of residence	Town with <20,000 residents or village	161	19.0
	City with 20,000–100,000 residents	510	60.1
	City with >100,000 residents	177	20.9

**Table 2:** The Acne Quality of Life (AQoL) Scale detailed results accompanied by the Social Quality of Life (SOCQOL) Score in the group of Polish female adolescents participating in the study.

Acne Quality of Life (AQoL) Scale		Mean ± SD	Median (Min–Max)
AQoL Scale items	Not feeling self-conscious in the presence of others	0.75 ± 0.89	0.5 * (0–3)
	Decrease in socialization with others	0.47 ± 0.75	0.0 * (0–3)
	Difficulties in relationship with spouse/partner	0.26 ± 0.65	0.0 * (0–3)
	Difficulties in relationship with close friends	0.25 ± 0.61	0.0 * (0–3)
	Difficulties in relationship with immediate family	0.20 ± 0.56	0.0 * (0–3)
	Feeling like an “outcast” most of the time because of the effect of acne upon appearance	0.37 ± 0.76	0.0 * (0–3)
	People making fun of appearance	0.36 ± 0.72	0.0 * (0–3)
	Feeling rejected in romantic relationship because of the effect of acne upon appearance	0.22 ± 0.63	0.0 * (0–3)
	Feeling rejected by friends because of the effect of acne upon appearance	0.18 ± 0.54	0.0 * (0–3)
Social Quality of Life (SOCQOL) Score		0.34 ± 0.54	0.1 * (0–3)

* nonparametric distribution (the Shapiro–Wilk test; *p* ≤ 0.05).

**Table 3:** The Acne Disability Questionnaire (ADQ) detailed results accompanied by the Cardiff Acne Disability Index (CADI) in the group of Polish female adolescents participating in the study.

Acne Disability Questionnaire (ADQ)		Mean ± SD	Median (Min–Max)
ADQ items	Feeling aggressive, frustrated, or embarrassed	0.96 ± 0.94	1.0 * (0–3)
	Feeling interference in daily social life, social events, or relationships with members of the opposite sex	0.52 ± 0.80	0.0 * (0–3)
	Avoided public changing facilities or wearing swimming costumes	0.56 ± 0.93	0.0 * (0–3)
	Feelings about the appearance of skin	0.94 ± 0.84	1.0 * (0–3)
	How bad they think their acne is	0.88 ± 0.74	1.0 * (0–3)
Cardiff Acne Disability Index (CADI) total score		3.85 ± 3.27	3.0 * (0–15)

* nonparametric distribution (the Shapiro–Wilk test; *p* ≤ 0.05).

**Table 4:** The Acne-specific Food Frequency Questionnaire (Acne-FFQ) detailed results in the group of Polish female adolescents participating in the study.

	Serving Size for Acne-FFQ	Servings Per Week	
		Mean ± SD	Median (Min–Max)
Vegetables	80 g	6.69 ± 8.10	4.0 * (0–75.0)
Fruit	80 g	18.76 ± 92.91	4.0 * (0–1500.0)
Water	250 g	19.78 ± 26.85	9.0 * (0–500.0)
Milk	250 g	2.80 ± 9.66	1.0 * (0–250.0)
Other dairy beverages	250 g	1.77 ± 2.54	1.0 * (0–40.0)
White bread	35 g	6.59 ± 8.19	4.0 * (0–70.0)
Wholegrain bread	35 g	4.30 ± 5.78	2.0 * (0–50.0)
Other white cereal products	70 g	3.12 ± 3.18	2.0 * (0–42.0)
Other wholegrain cereal products	70 g	2.06 ± 2.45	1.0 * (0–30.0)
Fish	100 g	0.93 ± 1.56	1.0 * (0–30.0)
Fast foods	1 meal	1.37 ± 1.72	1.0 * (0–28.0)
Salty snacks	1 serving	1.91 ± 1.95	1.0 * (0–20.0)
Chocolate confectionary	1 serving	2.59 ± 2.70	2.0 * (0–30.0)
Other confectionary	1 serving	2.15 ± 2.04	2.0 * (0–17.0)

* nonparametric distribution (the Shapiro–Wilk test; *p* ≤ 0.05).

**Table 5:** The analysis of correlations between the consumption of food items within the Acne-specific Food Frequency Questionnaire (Acne-FFQ) and the acne-related quality of life, assessed based on the Social Quality of Life (SOCQOL) Score calculated from the Acne Quality of Life (AQoL) Scale results, as well as the Cardiff Acne Disability Index (CADI) calculated from the Acne Disability Questionnaire (ADQ) in the group of Polish female adolescents participating in the study.

	SOCQOL Score		CADI	
	*p*	R *	*p*	R *
Vegetables	0.3153	−0.0345	0.2853	−0.0367
Fruit	0.8276	−0.0075	0.8558	−0.0062
Water	0.1574	−0.0486	0.6423	−0.0160
Milk	0.2991	−0.0357	0.1580	−0.0485
Other dairy beverages	0.1957	−0.0445	0.0414	−0.0701
White bread	0.8982	0.0044	0.9624	0.0016
Wholegrain bread	0.4811	−0.0242	0.1631	−0.0479
Other white cereal products	0.7208	−0.0123	0.7038	0.0131
Other wholegrain cereal products	0.9545	−0.0020	0.6379	−0.0162
Fish	0.1047	0.0558	0.3705	0.0308
Fast foods	0.6402	0.0161	0.0450	0.0688
Salty snacks	0.1763	0.0465	0.0342	0.0727
Chocolate confectionary	0.0978	0.0569	0.0147	0.0837
Other confectionary	0.5790	0.0191	0.0770	0.0607

* the Spearman rank correlation coefficient (due to nonparametric distribution).

**Table 6:** The comparison of the consumption of food items within the Acne-specific Food Frequency Questionnaire (Acne-FFQ) in subgroups stratified by the acne-related quality of life, assessed based on the Acne Quality of Life (AQoL) Scale in the group of Polish female adolescents participating in the study.

	No Acne-Related Decrease in the Quality of Life (*n* = 367)		Acne-Related Decrease in the Quality of Life (*n* = 481)		*p* **
	Mean ± SD	Median (Min–Max)	Mean ± SD	Median (Min–Max)	
Vegetables	6.53 ± 7.73	4.0 * (0–70.0)	6.81 ± 8.36	4.0 * (0–75.0)	0.5441
Fruit	19.66 ± 102.25	4.0 * (0–4.0)	18.07 ± 85.20	4.0 * (0–1000.0)	0.9173
Water	20.93 ± 33.41	9.0 * (0–500.0)	18.90 ± 20.47	9.0 * (0–150.0)	0.7225
Milk	2.38 ± 3.52	1.0 * (0–28.0)	3.13 ± 12.45	1.0 * (0–250.0)	0.5560
Other dairy beverages	1.74 ± 2.10	1.0 * (0–16.0)	1.80 ± 2.84	1.0 * (0–40.0)	0.5955
White bread	6.69 ± 8.41	4.0 * (0–64.0)	6.53 ± 8.03	4.0 * (0–70.0)	0.5774
Wholegrain bread	4.42 ± 6.37	2.5 * (0–50.0)	4.21 ± 5.30	2.0 * (0–40.0)	0.7168
Other white cereal products	3.27 ± 3.54	2.0 * (0–42.0)	3.00 ± 2.86	2.0 * (0–31.0)	0.5525
Other wholegrain cereal products	2.12 ± 2.77	1.0 * (0–30.0)	2.01 ± 2.18	1.0 * (0–14.0)	0.9027
Fish	0.83 ± 1.11	0.5 * (0–12.0)	1.01 ± 1.83	1.0 * (0–30.0)	0.2214
Fast foods	1.40 ± 2.03	1.0 * (0–28.0)	1.34 ± 1.44	1.0 * (0–10.0)	0.8755
Salty snacks	1.81 ± 1.78	1.0 * (0–10.0)	2.00 ± 2.07	1.5 * (0–20.0)	0.1750
Chocolate confectionary	2.44 ± 2.55	2.0 * (0–20.0)	2.70 ± 2.81	2.0 * (0–30.0)	0.0877
Other confectionary	2.17 ± 2.06	2.0 * (0–15.0)	2.13 ± 2.04	2.0 * (0–17.0)	0.7652

* nonparametric distribution (the Shapiro–Wilk test; *p* ≤ 0.05); ** the Mann–Whitney *U* test (due to nonparametric distribution).

**Table 7:** The comparison of the consumption of food items within the Acne-specific Food Frequency Questionnaire (Acne-FFQ) in subgroups stratified by the acne-related quality of life, assessed based on the Acne Disability Questionnaire (ADQ) in the group of Polish female adolescents participating in the study.

	No Acne-Related Decrease in the Quality of Life (*n* = 135)		Acne-Related Decrease in the Quality of Life (*n* = 713)		*p* **
	Mean ± SD	Median (Min–Max)	Mean ± SD	Median (Min–Max)	
Vegetables	6.88 ± 8.60	4.0 * (0–65.0)	6.52 ± 7.72	4.0 * (0–75.0)	0.5898
Fruit	14.30 ± 86.18	4.0 * (0–1000.0)	19.61 ± 94.17	4.0 * (0–1500.0)	0.8838
Water	21.16 ± 25.10	9.0 * (1.0–126.0)	19.52 ± 27.18	9.0 * (0–500.0)	0.4667
Milk	2.79 ± 4.27	1.0 * (0–28.0)	2.81 ± 10.37	1.0 * (0–250.0)	0.2698
Other dairy beverages	1.94 ± 2.26	1.0 * (0–15.0)	1.74 ± 2.59	1.0 * (0–40.0)	0.2264
White bread	6.71 ± 8.78	4.0 * (0–45.0)	6.57 ± 8.08	4.0 * (0–70.0)	0.7926
Wholegrain bread	4.08 ± 5.13	3.0 * (0–28.0)	4.34 ± 5.90	2.0 * (0–50.0)	0.8610
Other white cereal products	3.06 ± 2.66	2.0 * (0–14.0)	3.13 ± 3.27	2.0 * (0–42.0)	0.6969
Other wholegrain cereal products	2.21 ± 2.75	1.0 * (0–16.0)	2.03 ± 2.39	1.0 * (0–30.0)	0.9513
Fish	0.95 ± 1.38	1.0 * (0–12.0)	0.93 ± 1.59	1.0 * (0–30.0)	0.8437
Fast foods	1.50 ± 2.90	1.0 * (0–28.0)	1.34 ± 1.39	1.0 * (0–10.0)	0.3754
Salty snacks	1.79 ± 1.80	1.0 * (0–10.0)	1.94 ± 1.98	1.0 * (0–20.0)	0.3190
Chocolate confectionary	2.52 ± 2.84	2.0 * (0–20.0)	2.60 ± 2.68	2.0 * (0–30.0)	0.5800
Other confectionary	2.15 ± 2.29	1.2 * (0–15.0)	2.15 ± 2.00	2.0 * (0–17.0)	0.5743

* nonparametric distribution (the Shapiro–Wilk test; *p* ≤ 0.05); ** the Mann–Whitney *U* test (due to nonparametric distribution).
